# The cross-national applicability of lean implementation measures and hospital performance measures: a case study of Finland and the USA

**DOI:** 10.1093/intqhc/mzab097

**Published:** 2021-06-24

**Authors:** Elina Reponen, Thomas G Rundall, Stephen M Shortell, Janet C Blodgett, Ritva Jokela, Markku MÄkijÄrvi, Paulus Torkki

**Affiliations:** Center for Lean Engagement and Research in Healthcare, School of Public Health, University of California, 50 University Hall, Berkeley, CA, 94720-7360, USA; HUS Helsinki University Hospital, P.O.Box 760, 00029 HUS, Finland; Center for Lean Engagement and Research in Healthcare, School of Public Health, University of California, 50 University Hall, Berkeley, CA, 94720-7360, USA; Center for Lean Engagement and Research in Healthcare, School of Public Health, University of California, 50 University Hall, Berkeley, CA, 94720-7360, USA; Center for Lean Engagement and Research in Healthcare, School of Public Health, University of California, 50 University Hall, Berkeley, CA, 94720-7360, USA; HUS Helsinki University Hospital, P.O.Box 760, 00029 HUS, Finland; HUS Helsinki University Hospital, P.O.Box 760, 00029 HUS, Finland; Department of Public Health, University of Helsinki, P.O.Box 20, Helsinki 00014, Finland

**Keywords:** cross-national lean benchmarking, health-care system model, benchmarking, lean healthcare, performance improvement, performance measures

## Abstract

**Background:**

Health-care organizations around the world are striving to achieve transformational performance improvement, often through adopting process improvement methodologies such as lean management. Indeed, lean management has been implemented in hospitals in many countries. But despite a shared methodology and the potential benefit of benchmarking lean implementation and its effects on hospital performance, cross-national lean benchmarking is rare. Health-care organizations in different countries operate in very different contexts, including different health-care system models, and these differences may be perceived as limiting the ability of improvers to benchmark lean implementation and related organizational performance. However, no empirical research is available on the international relevance and applicability of lean implementation and hospital performance measures. To begin understanding the opportunities and limitations related to cross-national benchmarking of lean in hospitals, we conducted a cross-national case study of the relevance and applicability of measures of lean implementation in hospitals and hospital performance.

**Methods:**

We report an exploratory case study of the relevance of lean implementation measures and the applicability of hospital performance measures using quantitative comparisons of data from Hospital District of Helsinki and Uusimaa (HUS) Helsinki University Hospital in Finland and a sample of 75 large academic hospitals in the USA.

**Results:**

The relevance of lean-related measures was high across the two countries: almost 90% of the items developed for a US survey were relevant and available from HUS. A majority of the US-based measures for financial performance (66.7%), service provision/utilization (100.0%) and service provision/care processes (60.0%) were available from HUS. Differences in patient satisfaction measures prevented comparisons between HUS and the USA. Of 18 clinical outcome measures, only four (22%) were not comparable. Clinical outcome measures were less affected by the differences in health-care system models than measures related to service provision and financial performance.

**Conclusions:**

Lean implementation measures are highly relevant in health-care organizations operating in the USA and Finland, as is the applicability of a variety of performance improvement measures. Cross-national benchmarking in lean healthcare is feasible, but a careful assessment of contextual factors, including the health-care system model, and their impact on the applicability and relevance of chosen benchmarking measures is necessary. The differences between the US and Finnish health-care system models is most clearly reflected in financial performance measures and care process measures.

## Introduction

Health-care organizations around the world are seeking to transform service delivery to ensure high-quality care and equitable access while simultaneously containing costs [1, 2]. Despite the differences in the health-care system models used in different countries, the transformational performance improvement methodologies health-care organizations are adopting to achieve their goals are similar. One of the most popular methodologies is lean management [3, 4].

Lean is a management philosophy originally developed at Toyota that has since spread within the automobile industry, to manufacturing in general, and, more recently, to service industries [4]. During the last 20 years, many health-care organizations have adopted the lean philosophy. In short, the core of the lean philosophy is to strive for organizational alignment and continuous improvement to maximize customer value and to minimize waste. Lean implementation strategies are highly variable across health-care organizations, and few organizations have reached maturity on their lean journey [5].

While context has been identified as an important factor in the lean transformation of health-care organizations [6, 7], its dimensions beyond the intra-organizational level have been little studied [8]. One of the main dimensions of context is the predominant way healthcare is financed and delivered: the health-care system model used. Several frameworks for classifying health-care system models exist [9–15]. Pure representations of the basic health-care system models are, however, rare and most countries have developed a unique model adapted from one of the basic models. As many health-care organizations around the world are seeking answers to similar problems using the same methodologies, benchmarking is an attractive method for identifying best practices. However, cross-national lean benchmarking is rare, perhaps discouraged by the uncertainty regarding the applicability and relevance of specific measures of lean implementation and hospital performance in different contexts. A recent systematic review identified only 22 articles reporting benchmarking outcomes of lean initiatives in healthcare [16]. Furthermore, the authors of the systematic review identify a lack of consensus on performance dimensions and metrics in health-care organizations that have adopted lean or related performance improvement methodologies and suggest a conceptual framework comprising four main dimensions: patients, employed and affiliated staff, costs, and service provision [16].

Some examples of relevant contextual factors include cultural beliefs about health and illness, licensing regulations and laws, the way health-care providers and organizations are paid for their services, the extent and nature of clinicians’ participation in managerial decision-making, and the role of labor unions. These factors may facilitate or inhibit crucial prerequisites of lean implementation such as the level of resources available for performance improvement work, clinical and non-clinical staff members’ willingness to commit to increasing customer value, leader and staff buy-in to performance improvement practices, and the hospital performance measures that are compiled and available for review. Additionally, the model for lean implementation may be more directly adaptable to private than to public health-care organizations [17]. These contextual factors may also affect the applicability and relevance of measures of lean implementation and hospital performance, including clinicians’ participation in lean practices, hospital profitability, patient outcomes and patient satisfaction, across different national settings. We aim to explore this issue by assessing the applicability and relevance of key measures of lean implementation and hospital performance across two countries with substantially different contexts: the USA and Finland.

### Case study

The scarcity of published reports on international benchmarking in lean healthcare highlights the need for more exploratory research, including case reports, in the area [16]. To explore the extent to which data and measures involved with lean implementation and hospital performance might be relevant and applicable for promoting greater international benchmarking of lean implementation and performance improvement results, we conducted an exploratory case study examining large academic hospitals that have implemented lean in two countries, the USA and Finland. Lean principles and techniques in healthcare are largely universal even though implementation strategies may vary depending on the local context. Furthermore, in both the USA and Finland, the quality of medical education and research is high, and medical and technological innovations are actively incorporated into care processes, resulting in excellent conditions for providing high-quality care. In both countries, large academic hospitals provide a wide range of specialized care for the most complex medical needs of patients in their area. Finland has a Beveridge-type health-care system model, whereas the health-care system model in the USA is fragmented. A comparison of the health-care systems in these two countries is presented in Table 1.

**Table 1:** Comparison of health-care systems in Finland and in the USA

	Finland	United States
Health-care system model	Beveridge-type health-care system model Public healthcare (all residents) Municipal primary care centers Specialized care in central/university hospitals Covered by tax funds Minimal patient fees/copays Additional health-care services Private sector Private insurance/out-of-pocket Occupational healthcare (82% of workforce) [22] Both private and public sector service providers Statutory services: preventative healthcare and occupational health risks (29% of plans) [22] Additional more comprehensive coverage (71% of plans) [22]	Fragmented health-care system model Medicaid (17.9% of population) [23] and employer-based healthcare Similar to the Bismarck model with the distinction that insurance companies are primarily for-profit Medicare (17.8% of population) [23] Bears resemblance to the NHI model with the government acting as the single payer Veterans Affairs (1.0% of population) [23] Aligns with the Beveridge model Uninsured (8.5% of population) [23] Out-of-pocket healthcare
Insurance	Public health-care coverage: all residents Additional private health insurance 27% of overall population [24] 8% of people in the lowest income bracket [25] 30% of people in the highest income bracket [25]	Public insurance (34.4% of population) [23] Private insurance employment-based (55.1% of population) [23] direct purchase (10.8% of population) [23]
Health-care expenditure 2018 (% GDP)	9.0 [26]	17.7 [27]

NHI, National Health Insurance.

Despite the differences in the health-care systems between the USA and Finland, large academic hospitals are highly complex health-care organizations with similar challenges and opportunities for implementing transformational improvement methodologies such as lean management. However, the applicability of hospital performance measures and the relevance of lean implementation measures on the international level have not been assessed. This gap in knowledge led to the following research question:

- How do the differences between the health-care system models in the USA and in Finland affect the applicability and relevance of measures of lean implementation and selected hospital performance measures?

Specifically, we aim to test the following hypotheses:

H1: There are no major differences between the USA and Finland in the relevance of selected survey items on lean implementation in large academic hospitals.

H2: Patient outcome measures are more applicable across health-care system models than measures related to service provision and financial performance.

## Methods

We identified a subset of 75 large (>400 beds) academic hospitals that responded to the 2017 National Survey of Lean/Transformational Performance Improvement in Hospitals (NSL). Additional data on the 75 large academic US hospitals came from three sources: the American Hospital Association (AHA), the Center for Medicare and Medicaid Services (CMS) and the Agency for Healthcare Research and Quality (AHRQ) databases. Matching data were acquired from the databases of the Hospital District of Helsinki and Uusimaa (HUS), Helsinki, Finland.

### The 2017 National Survey of Lean/Transformational Performance Improvement Methods in Hospitals

In 2017, the AHA fielded the NSL addressing the use of lean and related transformational performance improvement methodologies to 4500 short-term acute general medical/surgical and pediatric US hospitals on behalf of the Center for Lean Engagement and Research in Healthcare (CLEAR) at University of California, Berkeley. The NSL was completed by the Chief Medical Officer (CMO), Chief Transformation Officer or equivalent position in each hospital. The NSL comprised responder details (four items) and 59 questions addressing the implementation and maturity of lean or related transformational performance improvement approaches (Supplementary Material Table S1), including detailed items on model cells, general hospital policies and practices with regard to lean, Central Improvement Team, Daily Management System, tools and methods, lean training and staffing, and subjective measures of hospital performance.

### AHA, CMS and AHRQ data

CLEAR obtained details on hospital characteristics and financial performance measures from the AHA Annual Survey and the annual CMS Medicare Cost Report. Publicly reported data on service provision, clinical outcomes and patient satisfaction for the NSL participant hospitals came from the annual CMS Hospital Compare, the annual CMS MEDPAR, the annual CMS Hospital Service Area File and AHRQ databases [18]. We categorized the available measures into three groups: service provision, patient outcomes and financial performance. Service provision consists of two subdivisions: utilization and care processes. Patient outcomes comprise both clinical outcomes and patient experience. All data were from 2018 with the exception of three hospital characteristic items, five care process measures (2015) and eight clinical outcome measures (average over the time period 1 July 2015 to 30 June 2018). Supplementary Material Table S2 presents a detailed list of hospital characteristics and measures included in each category.

### Hospital district of Helsinki and Uusimaa (HUS)

Helsinki, the hospital district that operates Helsinki University Hospital, is the largest health-care organization in Finland with 25 000 employees serving a population of 2.2 million people. HUS provides specialized care for the permanent residents of 24 municipalities and has additional special and national responsibilities for advanced care and for severe and uncommon diseases. The characteristics of HUS and the sample of large US academic hospitals are presented in Table 2.

**Table 2:** Hospital characteristics in the US national sample hospitals and HUS, Finland 2018

	Large (>400 beds) academic hospitals in the USA (*N* = 75 unless noted)	HUS, Finland
Hospital characteristics		
In operation 12 full months to the end of the reporting period	Yes: 74 (100.0%) *N* = 74	Yes
Type of authority responsible for establishing policy concerning overall operation of the hospital	State 9 (12.0%) County 4 (5.3%) City 1 (1.3%) Hospital district or authority 5 (6.7%) Church 2 (2.7%) Other not-for-profit 54 (72.0%)	Hospital district
Core-based statistical area type	Metro: 75 (100.0%)	Metro
Primary care physicians per 1000 pop.	0.84 (0.30), 0.77^a^	0.65
Medical specialists per 1000 pop.	1.96 (1.33), 1.54^a^	0.26^b^
Surgeons per 1000 pop.	1.02 (0.75), 0.81^a^	0.26^b^
Medical school affiliation reported to the American Medical Association	Yes 73 (97.3%) No 2 (2.7%)	No
Critical access hospital	Yes 1 (1.33%) No 74 (98.7%)	Yes
Rural referral center	Yes 15 (20.0%) No 60 (80.0%)	No
Sole community provider	No: 75 (100.0%)	Yes
Center for Improvement in Healthcare Quality accreditation	No: 75 (100%)	No
Participation in a bundled payment program	Yes: 30 (41.7%) No: 42 (58.3%) *N* = 72	Yes (partial)
Total hospital beds	784.79 (420.82), 650	2823
ED	Yes: 73 (98.6%) No: 1 (1.4%)	Yes
% of hospital’s net patient revenue paid on a capitated basis	1.46 (5.55), 0.00 *N* = 70	0.0
% of hospital’s net patient revenue paid on a shared risk basis	4.59 (10.82), 0.00 *N* = 63	100
Hospital beds set up and staffed	769.67 (415.75), 640	2823
Number of direct patient care RN FTEs	1874.07 (1155.63), 1543 *N* = 68	9339.3
FTE hospital unit total personnel	7023.87 (4618.65), 5685	20 614.9
Total privileged physicians	1520.60 (1252.12), 1163.5 *N* = 72	2737

FTE, full time equivalent; pop., population; RN, registered nurse.

For the US hospitals, data are presented as mean (standard deviation), median for continuous variables and *N* (%) for categorical variables.

a 2015 (latest available).

b 2016 (latest available).

The same questionnaire originally used for the 2017 NSL in the USA was completed by the Senior Medical Officer at the Lean Development Unit of HUS in March 2019. One of the authors (E.R.) worked with HUS IT Management and experts at HUS Joint Authority Administration to obtain hospital characteristics and a parallel dataset of measures from HUS records that matched the available US data on service provision, patient outcomes and financial performance. All data were from 2018 with the exception of two hospital characteristic items (Supplementary Material Table S2).

### Relevance and applicability

The relevance of the NSL items and the applicability of financial performance, service provision and patient experience measures to HUS context were assessed by the CMO and the Senior Medical Officer at the Lean Development Unit on a five-tier scale: routinely reported, available, available with modifications, unavailable and inapplicable. For the clinical outcome measures, we referred to the technical specifications available from CMS and AHRQ to compare the match with the measures available from HUS in detail. A physician author (E.R.) conducted a detailed manual comparison of the International Classification of Diseases (ICD)-9 and ICD-10 codes as well as procedural codes and Diagnosis Related Groups (DRGs) covered by each CMS or AHRQ outcome measure compared to similar outcome measures from HUS. The goodness-of-match of the outcome measures from CMS/AHRQ and HUS was then categorized on a three-tier scale as highly comparable, moderately comparable or not comparable.

### Quantitative comparisons

We used descriptive statistics to compare hospital performance measures from HUS and the sample of 75 large (>400 beds) academic US hospitals. HUS financial performance measures were converted from Euro to USD using the exchange rate averages in 2018. Measures in all categories rated either unavailable, inapplicable or not comparable were excluded from the quantitative comparisons.

### Institutional Review Board approval

This study was reviewed and approved by the Institutional Review Boards (IRBs) of HUS and the University of California, Berkeley.

## Results

### Relevance of lean survey items

**Figure 1:**
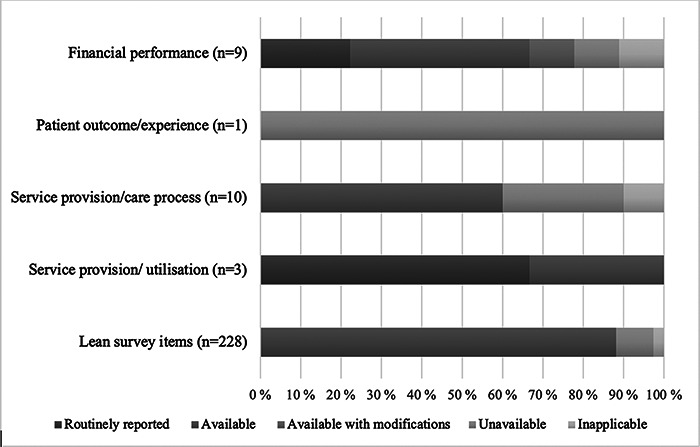
Relevance of lean survey items and applicability of hospital performance measures in HUS context.

The relevance assessment of the NSL items revealed a high applicability to the HUS context. While none of the 228 items collected through the survey were among those routinely reported at HUS, a vast majority of them (201, 88.2%) were available. Only 27 items (11.8%) were categorized as either unavailable or inapplicable (Supplementary Material Table S1). Of the 21 unavailable items, five were missing data and the other 16 were unanswered as irrelevant/unnecessary due to the questionnaire structure. The relevance of the lean survey items in HUS context is summarized in Figure 1.

### Applicability of hospital performance measures

The financial performance measures were moderately applicable to the HUS’s context: six measures (66.7%) were either routinely reported or available, whereas only two (22.2%) were unavailable or inapplicable to HUS’s context (Figure 1). All three measures of utilization in the service provision category were either routinely reported or available. None of the care process measures were routinely reported, but a majority (60.0%) were available. In the CLEAR dataset, the single patient experience measure was Hospital Consumer Assessment of Healthcare Providers and Systems (HCAHPS), which is not used in Finland and was thus categorized as unavailable.

The goodness-of-match assessment revealed that 14 of 18 (77.8%) clinical outcome measures were highly or moderately comparable. Only four clinical outcome measures (22.2%) were deemed not comparable due to major differences in coding or inclusion criteria. The detailed comparisons of clinical outcome measures are presented in Table 3.

**Table 3:** Comparability assessment of the clinical outcome measures

	US hospitals^a^	HUS, Finland^b^			Comparability		
Measure	Number of included codes	Number of included codes	% of CMS codes covered by HUS codes (2018)	% of HUS codes covered by CMS codes (2018)	High	Moderate	Not comparable
In-hospital mortality pneumonia	52	51	98.08 %	100.00 %	x		
Death rate in low-mortality DRGs	138^c^	135^d^	97.83 %	100.00 %	x		
Pressure ulcer rate	9	9	100.00 %	100.00 %	x		
30-day readmission rates	All-cause 30-day unplanned readmissions	All-cause 30-day unplanned readmissions	N/A	N/A	x		
In-hospital mortality AMI	13	10	76.92 %	100.00 %		x	
In-hospital mortality CHF	9	9	88.89 %	88.89 %		x	
In-hospital mortality stroke	32	28	87.50 %	100.00 %		x	
In-hospital mortality GI hemorrhage	58	27	N/A	N/A		x	
In-hospital mortality hip fracture	6	3	N/A	N/A		x	
Death rate among surgical inpatients with serious treatable conditions	116 dg codes 2 procedure codes	254 dg codes 3 procedure codes	N/A	N/A		x	
Mean 30-day risk-adjusted mortality heart failure	*8*	9	100.00 %	88.89 %		x	
Mean 30-day risk-adjusted mortality CABG		23	N/A	N/A		x	
Hip/knee arthroplasty complications of care	*36 index surgery codes* *105 complication dg codes* *1231 complication procedure codes*	15 index surgery codes 96 complication dg codes	N/A	N/A		x	
Hip/knee arthroplasty 30-day, unplanned readmission rates	*36 index surgery codes*		N/A	N/A		x	
Mean 30-day risk-adjusted mortality pneumonia	*28 primary discharge dg* *26 additional primary discharge dg if pneumonia as secondary dg*	51	100.00 %	54.90 %			x
Mean 30-day risk-adjusted mortality AMI	*5*	10	100.00 %	50.00 %			x
Mean 30-day risk-adjusted mortality COPD	*10 primary discharge diagnosis* *4 additional primary discharge diagnosis if combined with a secondary diagnosis of J440 or J441*	4	40.00 %	100.00 %			x
Mean 30-day risk-adjusted mortality stroke	*10*	28	100.00 %	35.71 %			x

CABG, Coronary Artery Bypass Graft; CHF, Congestive Heart Failure; COPD, Chronic Obstructive Pulmonary Disease; dg, diagnosis; GI, gastrointestinal; N/A, Not Applicable. ICD code comparisons at 3-digit level.

a Data sources AHRQ, CMS (Hospital compare); coding systems ICD-10, ICD-10CM and ICD-10-PCS.

b Data source HUS electronic medical records data, coding systems ICD-10/Nordic classification of surgical procedures.

c DRG groups, not ICD-10 codes.

d Matching Nord-DRG groups.

### Quantitative comparisons

Table 4 presents the quantitative comparisons between performance measures in the sample of large academic US hospitals and HUS. Of the care process measures, HUS performed above the US hospital sample mean in ischemic stroke patients who got medicine to break up a blood clot within 3 hours after symptoms started and median time (minutes) patients spent in the emergency department (ED) before being admitted as inpatients. On the other hand, median time (minutes) patients spent in the ED after the doctor decided to admit them as an inpatient before leaving the ED for their inpatient room and geometric mean length of stay were considerably longer in HUS compared to the US hospital sample mean.

**Table 4:** Comparisons of 2018 performance measures between the US national sample and HUS, Finland

	75 Large (>400 beds) academic US hospitals	HUS, Finland
Service provision, utilization		
Hospital unit admissions	36 279.76 (20 318.50), 32 379	197 690
Hospital unit inpatient days	207 721.19 (112 033.32), 176 062	258 926
Average daily census	581.33 (310–93), 492	709.39
Service provision, care process		
Ischemic stroke patients treated within 3 hours after symptoms started	97.67% (9.78), 95%^a^	99.90%
Median time (minutes) spent in ED, after decision to admit before leaving the ED for inpatient room	172.86 (86.40), 148.5^a^	547
Median time (minutes) spent in ED before being admitted as inpatient	401.10 (117.66), 389.5^a^	380
Median time (minutes) spent in ED before leaving (discharged patients)	208.96 (47.97), 204^a^	223
Percent of patients who left ED without being seen	3.00 (2.09), 2.50	5.29
Geometric mean length of stay	5.26 (0.71), 5.15^a^	43.98
Patient outcomes, clinical		
In-hospital mortality AMI (rate per 1000)	72.75 (29.99), 68.78	19.10
In-hospital mortality CHF (rate per 1000)	31.37 (12.61), 30.35	22.65
In-hospital mortality stroke (rate per 1000)	95.62 (40.52), 93.94	13.66
In-hospital mortality GI hemorrhage (rate per 1000)	26.82 (12.99), 24.39	14.53
In-hospital mortality hip fracture (rate per 1000)	26.32 (29.88), 21.28	11.86
In-hospital mortality pneumonia (rate per 1000)	27.61 (14.42), 25.89	28.97
Death rate in low-mortality DRGs (rate per 1000)	0.90 (0.142), 0.00	2.36
Pressure ulcer rate (rate per 1000)	1.09 (1.03), 0.93	2.52
Death rate among surgical inpatients with serious treatable conditions (rate per 1000)	108.75 (54.89), 109.14^b^	28.64
Mean 30-day risk-adjusted mortality heart failure (%)	10.45 (1.77), 10.30^b^	9.31
Mean 30-day risk-adjusted mortality CABG (%)	2.79 (0.72), 2.70^b^	1.18
Hip/knee arthroplasty complications of care (%)	2.59 (0.51), 2.60	0.69
Hip/knee arthroplasty 30-day, unplanned readmission (%)	4.02 (0.49), 4.10	1.05
30-day readmission (%)	15.52 (0.69), 15.50^b^	14.05
Financial performance		
Adjusted inpatient expense per discharge (USD)	8473.17 (2259.17), 8186.01	11 307.23
Adjusted operating profit margin	6.32 (13.21), 4.57	0.00
Average cost per ED visit	460.78 (103.78), 441.89	430.7
EBITDA (million USD)	242.54 (39.03), 132.65	143.22
EBITDA margin (EBITDA/total operating revenue)	12.08 (12.36), 10.39	0.05
Hospital total expense, excluding bad debt (million USD)	1,514.93 (1,085.63), 1,133.76	2,182.35
Hospital unit payroll expenses (million USD)	568.50 (473.27), 449.14	1,455.92

CABG, Coronary Artery Bypass Graft; CHF, Congestive Heart Failure; COPD, Chronic Obstructive Pulmonary Disease; GI, gastrointestinal.

For the US national sample hospitals, data are presented as mean (standard deviation), median for continuous variables and *N* (%) for categorical variables.

a Figure represents an average over period 1 July 2015 to 30 June 2018.

b 2015 (latest available).

Comparisons of financial performance metrics show that the adjusted inpatient expense per discharge, hospital unit payroll expenses, and the total hospital expense excluding bad debt in HUS exceeded the US hospital sample means. Average cost per ED visit in HUS, adjusted operating profit margin, and Earnings Before Interest Taxes Depreciation and Amortization (EBITDA), and EBITDA margin were below the US hospital sample mean.

In the quantitative comparisons of clinical outcome measures, HUS outperformed the US hospital sample median in all measures except in-hospital mortality rate for pneumonia, death rate in low-mortality DRGs and pressure ulcer rate. HUS’s performance advantage was largest in in-hospital mortality for acute myocardial infarction (AMI; fourfold), death rate among surgical inpatients with serious treatable conditions (fourfold) and in-hospital mortality for stroke (sevenfold).

## Discussion

### Statement of principal findings

Our finding that almost 90% of the lean survey items originally designed for US hospitals were available and relevant in HUS supports our first hypothesis and suggests that issues related to the adoption and implementation of lean in these large academic hospitals are, to a large extent, similar. Thus, international comparisons of lean adoption strategies and implementation methods may not be significantly constrained by the differences in health-care models. A total of 77.8% of the clinical outcome measures were highly or moderately comparable, indicating that clinical outcome measures were less affected by the differences in health-care system models than measures related to service provision and financial performance, thus supporting our second hypothesis. Additionally, our quantitative comparisons showed that clinical outcomes data can be successfully used for international benchmarking given that the specifications for each measure are carefully matched.

### Strengths and limitations

The strengths of this study include the careful assessment of relevance and applicability of the benchmarking measures. Extensive cooperation between a physician author, HUS Joint Authority Administration experts and HUS IT Department staff was undertaken to ensure the closest match possible between HUS and US measures.

This study also has some limitations. Despite careful attention to measure specifications, relevance and applicability, differences caused by differences in reporting and coding systems cannot be excluded. The quantitative comparisons of 30-day mortality figures, death rate among surgical inpatients with serious treatable conditions, and 30-day readmissions between the sample of US hospitals and HUS should be interpreted with caution since the US hospital sample numbers represent an average over a 3-year time period whereas the HUS figures are for 2018 only. Furthermore, the case study compares only two countries and the results may not be directly applicable to comparisons with other countries. The large difference in the numbers of hospitals in Finland and in the USA compared in the case study is also a limitation. While limited data availability and the limited number of academic medical centers in Finland prevented a more even comparison, this is an exploratory case study into a previously unexplored field and we hope it sparks interest in further research.

### Interpretation within the context of the wider literature

Few studies reporting cross-national comparisons of lean performance improvement initiatives in health-care organizations have been published to date [19, 20]. These studies report on financial and service provision measures and provide basic contextual data such as location and some hospital characteristics. However, the relevance and applicability of measures used for comparisons across contexts are not discussed in detail [19, 20]. In particular, care processes are significantly impacted by requirements unique to health-care system models, and unsurprisingly the applicability of these measures across the two countries in our case study was lower than that in the other performance categories.

Reports of international comparisons of patient outcomes in lean healthcare are lacking. The clinical outcome measures routinely reported in US hospitals showed good applicability to HUS: only four measures were not comparable. This together with the findings of our quantitative comparisons indicates that clinical outcomes data can be successfully used for international benchmarking given the specifications for each measure are carefully matched. Unfortunately, the US data included only one measure of patient experience, the HCAHPS score, which is not used in Finland, thus preventing further comparisons of this dimension.

Performance measures related to utilization of services were most likely to be applicable in both the USA and Finland, suggesting the importance of these measures in both contexts. Utilization measures are highly dependent on the size and patient volume of a hospital, and the quantitative comparisons may reflect that HUS is considerably larger than the mean of the academic large US hospitals included in the sample. According to Organization for Economic Cooperation and Development (OECD) data, the average length of hospital stay in Finland is longer than that in the USA (6.4 days vs. 5.5 days, respectively) [21]. The relatively large difference in geometric mean length of stay may result from differences in the organization of care delivery, for example the availability of post-discharge care and the range of services provided by the hospitals in the US sample and HUS. Similarly, the percentage of patients who left the ED unseen may be highly dependent on the local care processes and the availability of primary care clinics providing urgent care after hours. Among the financial measures, adjusted operating profit margin and EBITDA margin are relevant and available both in US hospitals and in Finland, but quantitative comparisons are complex due to the differences in financing systems: HUS’s profits are returned to the 24 member municipalities at the end of each fiscal year.

### Implications for policy, practice and research

Our results are encouraging for international benchmarking of research findings in lean healthcare. The USA–Finland comparisons are merely a starting point. More comparative research with multiple organizations representing different health-care models is needed to further investigate the impact of differences in health-care systems. Many health-care organizations across the world are adopting lean, but most lean health-care research still originates in pioneering countries such as the USA. Thus, health-care managers and operational leaders may need to look beyond their own country for research evidence to identify best practices and benchmark performance. Our results indicate that such cross-national comparisons are feasible.

## Conclusions

The differences between the health-care system models in the USA and in Finland do not limit the applicability and relevance of measures of lean implementation, whereas comparisons of hospital performance measures warrant careful attention to the context. Our exploratory case study comparing large academic hospitals in two different health system model contexts—the USA and Finland—illustrates that the NSL measures are highly relevant and available from health-care organizations operating in countries outside the USA. The applicability of clinical patient outcome measures seems to be less affected by differences in health-care system models than the applicability of service provision measures and financial performance measures.

## Supplementary Material

mzab097_Supp

## Data Availability

Data are available within the article or its supplementary materials.
