# Supplementary material for: The cross-national applicability of lean implementation measures and hospital performance measures: a case study of Finland and the USA
Source: Int J Qual Health Care. 2021 Jun 24;33(3):mzab097. doi: 10.1093/intqhc/mzab097 (PMC8886912; doi:10.1093/intqhc/mzab097)
Supplement: mzab097_Supp [file mzab097_Supp.zip › Supplementary table 1 R1.docx]

| Supplementary table 1. The relevance of the items in the 2017 National Survey of Lean/Transformational Performance Improvement Methods in XX University Hospital, Finland. | | | | |
| --- | --- | --- | --- | --- |
| Survey questions | | **Available items (n)** | **Unavailable items (n)** | **Inapplicable items (n)** |
| *Overview* | |  |  |  |
|  | Is your hospital currently engaged in any of the following transformational performance improvement approaches? (9 options including Lean without Six Sigma, Lean Six Sigma combined, and Robust Performance Improvement) | 8 | 1 | 0 |
|  | Which, if any, of the following transformational performance improvement approaches is the primary approach used in your hospital to improve quality, safety, efficiency, and patient satisfaction? Please select only one. (10 options including Lean without Six Sigma, Lean Six Sigma combined, and Robust Performance Improvement) | 1 | 0 | 0 |
|  | When did you first begin implementing Lean? (Month and year) | 1 | 1 | 0 |
|  | Which of the statements below best describes the approach used in the implementation? Please select only one. (6 options ranging from “We implemented some lean management elements in a single department” to “ We implemented a comprehensive Lean daily management system hospital-wide including all or nearly all departments”) | 1 | 0 | 0 |
|  | At this point in time, which of the statements below best describes your hospital’s journey towards overall transformational performance improvement? Please select only one. (4 options ranging from “We are still in the new start-up stage” to “We have become a mature transformational performance improvement hospital”) | 1 | 0 | 0 |
|  | Which of the following hospital units are currently using the principles and tools of Lean? (A list of 28 departments and “Other, please indicate”) | 23 | 0 | 6 |
|  | At this point in time, how many Lean projects are currently being implemented in your hospital? (4 categories: 1-3, 4-6, 7-9, and 10+) | 1 | 0 | 0 |
| *Model cells* | |  |  |  |
|  | Did your hospital initiate its use of Lean with one or more model cells? Please respond yes or no. | 1 | 0 | 0 |
|  | Did Lean implementation in your hospital start with one model cell, or was it deployed across multiple model cells at the same time? Please select one. (2 options: started in one or multiple model cells) | 1 | 0 | 0 |
|  | How many active model cells does your hospital currenly have? (Whole number between 0 and 100) | 1 | 0 | 0 |
|  | Using your best judgement, please indicate the name of the unit that is the model cell that is furthest along in implementing Lean philosophy and management practices (the model cell having the broadest acceptance of the Lean philosophy and the most consistent use of Lean tools and practices in comparison to other model cells at this time). Please select one. (A list of 30 departments and “Other, please indicate”) | 1 | 0 | 0 |
|  | When was Lean first started in the model cells? (Month and year) | 1 | 1 | 0 |
|  | In what ways, if any, are **doctors** ROUTINELY involved in the model cell’s performance improvement efforts? Please respond Yes or No to involvement in each of the following Lean improvement efforts (5 activities, including “Coaching” and “Attending daily huddles”, and “Other, please indicate”) | 5 | 1 | 0 |
|  | In what ways, if any, are **nurses** ROUTINELY involved in the model cell’s performance improvement efforts? Please respond Yes or No to involvement in each of the following Lean improvement efforts (5 activities, including “Coaching” and “Attending daily huddles”, and “Other, please indicate”) | 5 | 1 | 0 |
|  | In what ways, if any, are **department managers** ROUTINELY involved in the model cell’s performance improvement efforts? Please respond Yes or No to involvement in each of the following Lean improvement efforts (5 activities, including “Coaching” and “Attending daily huddles”, and “Other, please indicate”) | 5 | 1 | 0 |
|  | How often do the following people actively participate in the Lean model cell team’s performance improvement efforts? A list of 7 positions ranging from the “Chief Executive Officer” to “Patients”, scale “Always”, “Sometimes”, or “Never”) | 7 | 0 | 0 |
|  | To what extent are model cell team members able to write standard work that is clear, useful, and accepted by the people who use it? Please select one. (4 options ranging from “Not at all” to “Very able”) | 1 | 0 | 0 |
|  | To what extent is the model cell’s standard work tied to the hospital’s overall goals and metrics? Please select one. (4 options ranging from “Not at all” to “Very tied”) | 1 | 0 | 0 |
|  | Has the hospital taken any steps to spread the design of the model cell to other hospital units? Please respond Yes or No. | 1 | 0 | 0 |
|  | If you answered NO to question 23 (the previous question), why not? Please respond Yes or No to each of the following items. (A list of 10 items ranging from “Unsatisfactory results from current model cell improvement work” to “Leadership not currently prioritizing spread of model cell framework due to other management concerns”, and “Other, please indicate”) | 0 | 11 | 0 |
| *General hospital policies and practices* | |  |  |  |
|  | Please select to what extent you AGREE or DISAGREE with the following statements. (8 statements, e.g. “The outcomes desired from using the Lean approach are clear, widely understood, and shared” and “The hospital’s leaders set benchmarks to assess progress with Lean initiatives”, scale “Strongly disagree”, “Disagree”, “Neither agree nor disagree”, “Agree”, or “Strongly agree”) | 8 | 0 | 0 |
|  | Has your hospital developed a vision for its future that is used as the “True North” for its Lean transformation? Yes/No | 1 | 0 | 0 |
|  | Please respond to the following statements about your hospital’s rewards and recognition related to performance improvement (PI). Please respond Yes or No. (3 options ranging from “Departments give recognition and/or rewards to individuals and teams for PI success” to “External organizations give the hospital recognition and/or rewards for PI success”) | 3 | 0 | 0 |
| *Central Improvement Team* | |  |  |  |
|  | Does your hospital or the system to which your hospital belongs have a Central Improvement Team or equivalent group? Please respond Yes or No. | 1 | 0 | 0 |
|  | How many people are on the Central Improvement Team? (Categories 1-5, 6-10, 11-15, and 16+) | 1 | 0 | 0 |
|  | Which of the following are the Central Improvement Team’s functions? Please respond Yes or No to each function (4 options including “Facilitate Lean activities” and “Prioritize improvement projects”, and “Other, please indicate”) | 4 | 1 | 0 |
|  | How many Lean facilitators (project support staff with advanced training in Lean that support Lean initiatives are on the Central Improvement Team? (Categories 0, 1-5, 6-10, 11-15, 16+) | 1 | 0 | 0 |
|  | Does the Central Improvement Team include leaders from any of the following departments? Please respond Yes or No for each department. (List of 3 departments: Finance, Human Resources, and Information Technology) | 3 | 0 | 0 |
|  | To whom does the Central Improvement Team report? Please select one. (5 options ranging from “Chief Executive Officer” to “Chief Medical Officer”, and “Other, please indicate”) | 1 | 0 | 0 |
|  | Is your hospital currently using an outside consultant to assist in Lean implementation (including training)? Please respond Yes or No. | 1 | 0 | 0 |
|  | How long have you been using this consultant? Please select one (7 categories ranging from “1-6 months” to “more than 36 months”) | 1 | 0 | 0 |
|  | If you responded NO to question 34 (Is your hospital currently using an outside consultant), has the hospital used a consultant to assist with Lean implementation (including training) in the past? Please respond Yes or No. | 0 | 1 | 0 |
| *Daily Management System* | |  |  |  |
|  | Which of the following activities do **C-suite leaders** ROUTINELY do? Please respond Yes or No to each of the following activities. (A list of 12 activities including “Daily huddles”, “Go on Gemba walks”, and “Practice A3 thinking”) | 12 | 0 | 0 |
|  | Which of the following activities do **VPs and Division Heads** ROUTINELY do? Please respond Yes or No to each of the following activities. (A list of 12 activities including “Daily huddles”, “Go on Gemba walks”, and “Practice A3 thinking”) | 12 | 0 | 0 |
|  | Which of the following activities do **Managers** ROUTINELY do? Please respond Yes or No to each of the following activities. (A list of 12 activities including “Daily huddles”, “Go on Gemba walks”, and “Practice A3 thinking”) | 12 | 0 | 0 |
|  | Please select how often the following statements are true in your hospital. (2 statements, e.g. “Our hospital’s senior leadership team is able to rapidly identify waste and process failures and address them”, scale “Never”, “Some of the time”, “Most of the time”, and “All of the time”) | 2 | 0 | 0 |
|  | Please select to what extent you AGREE or DISAGREE with the following statements. (5 statements, e.g. “Our hospital’s senior leadership team makes data-driven decisions”, and “Our hospital’s senior leadership team spends time with the caregivers to understand barriers to patient care”, scale “Strongly disagree”, “Disagree”, “Neither agree nor disagree”, “Agree”, or “Strongly agree”) | 5 | 0 | 0 |
| *Tools and Methods* | |  |  |  |
|  | To what extent has your hospital implemented the following tools and methods? ( A list of 14 tools/methods including “Daily huddles”, “mistake-proofing”, and “5S: redesign of physical work space to improve efficiency”, and “Other, please indicate”) | 14 | 1 | 0 |
| *Finance* | |  |  |  |
|  | Please select the extent to which you AGREE or DISAGREE with the following statements. (3 statements, e.g. “Our organization’s finance department is an important partner in achieving our Lean goals and objectives”, scale “Strongly disagree”, “Disagree”, “Neither agree nor disagree”, “Agree”, or “Strongly agree”) | 3 | 0 | 0 |
|  | Does your finance department prepare forecasts for managers? Yes or No. | 1 | 0 | 0 |
|  | How frequently does your finance department provide frontline managers with forecasts of revenues and expenditures? Please select one. (4 options ranging from “Monthly” to “Annually”, and “Other, please indicate”) | 1 | 0 | 0 |
|  | Does your finance department use visual management (such as A3 sheets, trend charts, or storyboards in doing its work and communicating with each other? Please respond Yes or No. | 1 | 0 | 0 |
| *Human Resources* | |  |  |  |
|  | Please select the extent to which you AGREE or DISAGREE with the following statements. (5 statements, e.g. “Our HR team’s primary role is to act as advisors to managers”, scale “Strongly disagree”, “Disagree”, “Neither agree nor disagree”, “Agree”, or “Strongly agree”) | 5 | 0 | 0 |
|  | Has your hospital declared an official **No Layoff** policy as it relates to Lean? Please respond Yes or No | 1 | 0 | 0 |
|  | Are there labor unions in your hospital? Please respond Yes or No. | 1 | 0 | 0 |
|  | Do the labor unions in your hospital support Lean? Please select one. (Three options ranging from “Our hospital’s labor unions generally support Lean” to “Our hospital’s labor unions generally oppose Lean”) | 1 | 0 | 0 |
|  | Please select the extent to which you AGREE or DISAGREE with the following statements. (3 statements, e.g., “The people in the hospital are well trained in Lean management philosophy and principles”, scale “Strongly disagree”, “Disagree”, “Neither agree nor disagree”, “Agree”, or “Strongly agree”) | 3 | 0 | 0 |
|  | Does your human resources department use visual management (such as A3 sheets, trend charts, or storyboards in doing its work and communicating with each other? Please respond Yes or No. | 1 | 0 | 0 |
| *Information Technology* | |  |  |  |
|  | Please select to which extent you AGREE or DISAGREE with the following statements. (6 statements, e.g. “Our hospital’s IT department provides managers with the data and analysis they need to achieve their goals”, scale “Strongly disagree”, “Disagree”, “Neither agree nor disagree”, “Agree”, or “Strongly agree”) | 6 | 0 | 0 |
|  | Does your IT department use visual management (such as A3 sheets, trend charts, or storyboards in doing its work and communicating with each other? Please respond Yes or No. | 1 | 0 | 0 |
| *Lean Training and Staffing* | |  |  |  |
|  | Approximately, what percentage of your hospital’s **managers** have been trained in scientific approaches to problem solving, such as the use of Plan-Do-Study-Act (PDSA) cycles? (5 categories ranging from “0%” to “75-100%”) | 1 | 0 | 0 |
|  | Approximately, what percentage of your hospital’s **nurses** have been trained in scientific approaches to problem solving, such as the use of Plan-Do-Study-Act (PDSA) cycles? (5 categories ranging from “0%” to “75-100%”) | 1 | 0 | 0 |
|  | Approximately, what percentage of your hospital’s **doctors** have been trained in scientific approaches to problem solving, such as the use of Plan-Do-Study-Act (PDSA) cycles? (5 categories ranging from “0%” to “75-100%”) | 1 | 0 | 0 |
|  | At this point in time, how many FTE employees in your hospital are **exclusively dedicated** to quality improvement? (7 categories from “0” to “26 or more”) | 1 | 0 | 0 |
|  | Please select the answer choice that best represents your hospital’s leadership training related to Lean performance improvement (PI). Please select one. (5 options ranging from “Initial training is/has been offered to senior leaders” to “PI training is a prerequisite for many leadership positions”) | 1 | 0 | 0 |
|  | Please select the answer choice that best represents your hospital’s staffing related to performance improvement (PI). Please select one. (5 options ranging from “No performance improvement staffing plan has been identified” to “PI-trained staff members are leading **most** improvement initiatives” | 1 | 0 | 0 |
| *Hospital Performance* | |  |  |  |
|  | Please respond to the following statements about your hospital’s performance improvement initiatives. (3 statements, e.g. “Our hospital realizes a positive return on investment from our performance improvement initiatives, response options “Yes”, “No”, and “Don’t know”) | 3 | 0 | 0 |
|  | Please indicate the extent to which you AGREE or DISAGREE with the following statements. (4 statements, e.g. “If patients are unhappy with the quality of services, immediate action is taken”, scale “Strongly disagree”, “Disagree”, “Neither agree nor disagree”, “Agree”, or “Strongly agree”) | 4 | 0 | 0 |
|  | What, if any, performance achievements in your organization can be primarily attributed to implementation of Lean? (15 options including “Reduced expenditures in two or more departments” and “Increased throughput in the emergency department”, and “Other significant achievement, please indicate as many as you like”) | 15 | 1 | 0 |
