# Supplementary material for: The cross-national applicability of lean implementation measures and hospital performance measures: a case study of Finland and the USA
Source: Int J Qual Health Care. 2021 Jun 24;33(3):mzab097. doi: 10.1093/intqhc/mzab097 (PMC8886912; doi:10.1093/intqhc/mzab097)
Supplement: mzab097_Supp [file mzab097_Supp.zip › Supplementary table 2 R1.docx]

**Supplementary Table 2. Case study measures by category and time period for data (full year unless otherwise indicated)**

| Hospital characteristics | Time period for US hospital sample data | Time period for HUS data |
| --- | --- | --- |
| Was the hospital in operation 12 full months to the end of the reporting period? | 2018 | 2018 |
| Type of authority responsible for establishing policy concerning overall operation of the hospital | 2018 | 2018 |
| Core-based statistical area type | 2018 | 2018 |
| Primary care physicians per 1,000 pop. | 2015 | 2018 |
| Medical specialists per 1,000 pop. | 2015 | 2016 |
| Surgeons per 1,000 pop. | 2015 | 2016 |
| Medical school affiliation reported to American Medical Association | 2018 | 2018 |
| Critical access hospital | 2018 | 2018 |
| Rural Referral Center | 2018 | 2018 |
| Sole Community Provider | 2018 | 2018 |
| Center for Improvement in Healthcare Quality accreditation | 2018 | 2018 |
| Does your hospital participate in a bundled payment program? | 2018 | 2018 |
| Total hospital beds (calculated field: sum of all individual bed counts) | 2018 | 2018 |
| Emergency department | 2018 | 2018 |
| What % of hospital's net patient revenue is paid on a capitated basis? | 2018 | 2018 |
| What % of the hospital's net patient revenue is paid on a shared risk basis? | 2018 | 2018 |
| Hospital beds set up and staffed | 2018 | 2018 |
| Number of direct patient care RN FTEs | 2018 | 2018 |
| Full time equivalent hospital unit total personnel | 2018 | 2018 |
| Total privileged physicians | 2018 | 2018 |
| Service provision: utilisation |  |  |
| Hospital unit admissions | 2018 | 2018 |
| Hospital unit inpatient days | 2018 | 2018 |
| Average daily census | 2018 | 2018 |
| Service provision: care processes |  |  |
| Ischemic stroke patients who got medicine to break up a blood clot within 3 hours after symptoms started (%) | 2015 | 2018 |
| Median time (minutes) patients spent in the emergency department, after the doctor decided to admit them as an inpatient before leaving the emergency department for their inpatient room | 2015 | 2018 |
| Median time (minutes) patients spent in ED before being admitted as Inpatient | 2015 | 2018 |
| Median time (minutes) patients spent in ED before leaving (emergency department arrival to emergency department departure for discharged emergency department patients) | 2015 | 2018 |
| Percent of patients who left ED without being seen (Patient left without being seen) | 2018 | 2018 |
| Geometric mean length of stay | 2015 | 2018 |
| Patient outcomes: clinical |  |  |
| In-hospital mortality AMI (rate per 1,000) | 2018 | 2018 |
| In-hospital mortality CHF (rate per 1,000) | 2018 | 2018 |
| In-hospital mortality Stroke (rate per 1,000) | 2018 | 2018 |
| In-hospital mortality GI hemorrhage (rate per 1,000) | 2018 | 2018 |
| In-hospital mortality hip fracture (rate per 1,000) | 2018 | 2018 |
| In-hospital mortality pneumonia (rate per 1,000) | 2018 | 2018 |
| Death rate in low-mortality Diagnosis Related Groups (DRGs) (rate per 1,000) | 2018 | 2018 |
| Pressure ulcer rate (rate per 1,000) | 2018 | 2018 |
| Death rate among surgical inpatients with serious treatable conditions (rate per 1,000) | average over 7/1/2015-6/30/2018 | 2018 |
| Mean 30-day risk-adjusted mortality heart failure (%) | average over 7/1/2015-6/30/2018 | 2018 |
| Mean 30-day risk-adjusted mortality pneumonia (%) | average over 7/1/2015-6/30/2018 | 2018 |
| Mean 30-day risk-adjusted mortality AMI (%) | average over 7/1/2015-6/30/2018 | 2018 |
| Mean 30-day risk-adjusted mortality COPD (%) | average over 7/1/2015-6/30/2018 | 2018 |
| Mean 30-day risk-adjusted mortality stroke (%) | average over 7/1/2015-6/30/2018 | 2018 |
| Mean 30-day risk-adjusted mortality CABG (%) | average over 7/1/2015-6/30/2018 | 2018 |
| Hip/knee arthroplasty complications of care (%) | 2018 | 2018 |
| Hip/knee arthroplasty 30-day, unplanned readmission rates (%) | 2018 | 2018 |
| 30-day readmission rates (%) | average over 7/1/2015-6/30/2018 | 2018 |
| Patient outcomes: experience |  |  |
| HCAHPS score (patient rating of overall hospital performance) | 2018 | N/A |
| Financial performance |  |  |
| Adjusted inpatient expense per discharge | 2018 | 2018 |
| Adjusted operating profit margin | 2018 | 2018 |
| Average cost per ED visit | 2018 | 2018 |
| Earnings before Interest Taxes Depreciation and Amortization (EBITDA) | 2018 | 2018 |
| EBITDA Margin (EBITDA / Total operating revenue) | 2018 | 2018 |
| Hospital total expense, excluding bad debt | 2018 | 2018 |
| Hospital unit payroll expenses | 2018 | 2018 |

Abbreviations: AMI, Acute Myocardial Infarction; CABG, Coronary Artery Bypass Graft; CHF, Congestive Heart Failure; COPD, Chronic Obstructive Pulmonary Disease; EBITDA, Earnings Before Interest Taxes Depreciation and Amortization; ED, Emergency Department, GI, gastrointestinal; HCAHPS, Hospital Consumer Assessment of Healthcare Providers and Systems; pop., population
